# A perspective for alzheimer disease from gut microbiota-associated NMR-based fecal metabolomics: a study with 5XFAD mice

**DOI:** 10.1007/s11011-026-01848-2

**Published:** 2026-04-24

**Authors:** Nazlıhan Tekin, Furkan Şahin, Betül Şahin, Zelal Zuhal Kaya, Ali Yılmaz, Stewart F Graham, Mustafa Serteser, Ahmet Tarık Baykal

**Affiliations:** 1 Acibadem Labmed Clinical Laboratories, Istanbul, Turkey; 2 Department of Biochemistry and Molecular Biology, Graduate School of Health Sciences, Acibadem Mehmet Ali Aydinlar University, Istanbul, Turkey; 3 Department of Medical Biology, Faculty of Medicine, Bahcesehir University, Istanbul, Turkey; 4 Department of Medical Biochemistry, Faculty of Medicine, Nisantasi University, Istanbul, 34398 Turkey; 5 Metabolomics Department, Corewell Health Research Institute, Royal Oak, MI 48073 USA; 6 Corewell Health William Beaumont University Hospital, Royal Oak, MI 48073 USA; 7 Oakland University-William Beaumont School of Medicine, Rochester, MI 48309 USA; 8 Department of Medical Biochemistry, School of Medicine, Acibadem Mehmet Ali Aydinlar University, Istanbul, 34450 Turkey

**Keywords:** Fecal metabolites, Alzheimer's disease, Nuclear magnetic resonance spectroscopy, 5XFAD mice

## Abstract

Alzheimer’s disease (AD), a neurodegenerative, irreversible, and progressive brain disorder, is characterized by memory loss and cognitive dysfunction. In neurodegeneration, gut microbiota and their metabolites have an impact on behavior and brain function. This study aimed to identify fecal candidate discriminant metabolites in 5XFAD mice assosciated with AD as compared to an age-matched wild-type (WT) healthy mice groups at three different time points (3, 6, and 9 month) using Proton Nuclear Magnetic Resonance (^1^H NMR) spectroscopy. A total of 18 male mice (*n* = 9 for 5XFAD mice and *n* = 9 for WT, with three animals per age group) were used to determine the fecal candidate discriminant metabolites. As a result of the study, it was identified a total of 67 metabolites in fecal samples. Among these, 19 metabolites were found significantly up-regulated or down-regulated in 5XFAD mice compared to WT mice during the disease progress. According to the volcano plot analysis (FC ≥ 1.2 and *p*-value ≤ 0.05), the potential candidate discriminant metabolites were fumarate and malate in 3-month-old 5XFAD mice, 3-methylxanthine, glucose, cholate, phenylacetate, and trimethylamine N-oxide (TMAO) in 6-month-old 5XFAD mice and cholate and succinate in 9-month-old 5XFAD mice. These findings highlight the potential of fecal metabolomics as a non-invasive exploratory approach for understanding AD disease mechanisms and progression. Furthermore, to the best of our knowledge, this pilot study is the first to utilize NMR spectroscopy to investigate age-stratified cross-sectional changes in fecal metabolites in 5xFAD mice, thereby advancing our understanding of disease progression and potentially informing future therapeutic strategies.

**Graphical Abstract:**

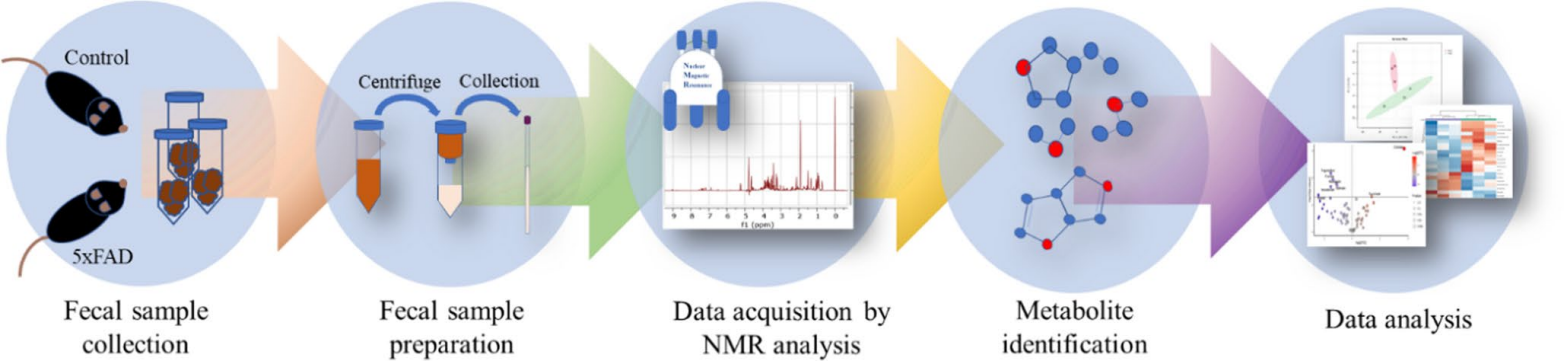

**Supplementary Information:**

The online version contains supplementary material available at 10.1007/s11011-026-01848-2.

## Introduction

Alzheimer’s disease (AD), a neurodegenerative, irreversible, and progressive brain disease, is characterized by memory loss and cognitive dysfunction. According to the Alzheimer’s Disease International (ADI), over 55 million people had dementia worldwide in 2020. It is estimated that this number will reach 78 million by 2030 and 139 million by 2050. Additionally, the annual global cost of dementia is expected to rise to US$ 2.8 trillion by 2030 (Alzheimer’s Disease International 2025). The progression of AD is known to be associated with tangles of hyperphosphorylated tau protein and the accumulation of amyloid beta (Aβ) (Spencer et al. 2013). Among the other hypotheses in AD pathogenesis, the amyloid cascade hypothesis is the most dominant. In this model, Aβ accumulation arises from aberrant proteolytic processing of amyloid precursor protein (APP) by secretases such as γ-secretase, β-secretase, or α-secretase (Delport and Hewer 2022), and the levels of Aβ and related enzyme activities are significantly higher in persons with AD than in healthy persons. Three stages as preclinical, mild cognitive impairment, and dementia are commonly observed in the progression of AD (Tahami Monfared et al. 2022). Given the high risk of progression to AD, individuals with mild cognitive impairment (MCI) would greatly benefit from enhanced prognosis and early intervention strategies, which could potentially slow disease progression and improve outcomes. Despite considerable research efforts, there is currently no effective treatment for AD, and its pathophysiology is still elusive (Wu et al. 2021). Numerous Aβ-targeted clinical trials and drug development efforts have been conducted over the past decades for the treatment of AD, and various therapeutic strategies continue to be explored. Nevertheless, due to the complexity and chronic nature of the disease, a definitive and effective treatment strategies has yet to be established (Sun et al. 2022).

Recent studies on AD have identified numerous risk factors that may contribute to the onset and progression of the disease, including genetic predisposition, age, educational level, lifestyle, diet, and gut health (Upadhyay and Gupta 2023). The human gut microbiome plays a significant role in influencing host physiology, including cognition, immune function, and behavior, highlighting the intricate gut-brain axis and its potential implications for overall health and disease (Lu et al. 2024). The recent clinical and translational studies have shown the alterations in fecal microbiota, metabolites, and metabolic pathways in AD compared to healthy control. Microbial metabolites, including tryptophan derivatives, bile acids, short-chain fatty acids (SCFAs), amino acid neurotransmitters, and catecholamines, can modulate the gut-brain axis. These metabolites can influence neurotransmission and disease pathogenesis, providing a functional link between the gut microbiome and the brain. These metabolites may play crucial roles in influencing neurotransmission, behavior, and disease development, effectively bridging the gut microbiota and brain function (Kim et al. 2021, 2022; Sun et al. 2022; Zhao et al. 2025). Moreover, preclinical and human studies on microbiota modulation through fecal transplantation and oral bacteriotherapy have demonstrated anti-inflammatory and antioxidant effects, increased plasma concentrations of neuroprotective hormones, restorated impaired proteolytic pathways, improved energy homeostasis with a corresponding reduction in AD molecular hallmarks, and improved behavioral and cognitive abilities by not using drugs (Bonfili et al. 2021). In this context, the gut-brain axis analysis was evaluated as a new promising approach for examining the biological and physiological mechanisms of AD (Upadhyay and Gupta 2023).

By leveraging metabolomics, researchers can comprehensively profile metabolites that play pivotal roles in the gut-brain axis, ultimately facilitating the discovery of novel biomarkers for disease diagnosis and therapeutic monitoring. This can potentially significantly advance our understanding of disease mechanisms and thus help to develop novel treatment modalities (Zhang et al. 2024). Nuclear Magnetic Resonance (NMR) and Mass Spectroscopy (MS) are the two main analytical methods to perform metabolomics and lipidomics studies (Nielsen et al. 2021). NMR has the advantageous qualities of being quantitative, high-throughput, and highly reproducible (Vignoli and Tenori 2023). In addition, NMR provides benefits such as little or no complex sample preparation, no need for chemical derivatization, and being unbiased because it does not rely on ionization strength (Wishart et al. 2022). Therefore, NMR can be a unique tool to identify metabolic variety in different biological samples (blood derivatives, cerebrospinal fluid, urine, saliva, fecal, and tissues).

Fecal metabolomics provides a non-invasive approach for understanding disease mechanisms and progression, reflecting the metabolic output of the gut microbiome. Although direct brain analysis provides indispensable information, the metabolomics of this tissue can only be performed invasively and yield accurate results postmortem (especially in humans) and in the advanced stages of the disease. However, because fecal samples can be obtained non-invasively and are reproducible (Wingfield et al. 2019; Órdenes et al. 2024), they allow for longitudinal studies within the same individual. Thus, changes in the fecal microbiome and its metabolites can be evaluated as biomarkers in the early stages of AD, unlike biomarkers that appear in brain tissue in the late stages of AD.

Primary objective of the current study is to employ NMR-based metabolomics to identify distinct fecal candidate discriminant metabolites in 5XFAD mice with AD compared to WT healthy control. Through this pilot study, by age-stratified cross-sectional investigating of fecal metabolic profiles, we seek to uncover potential microbial metabolites associated with AD progression, ultimately exploratively enhancing early detection and understanding of the disease. This approach has potential to provide novel insights into the underlying mechanisms of AD, opening avenues for novel therapeutic strategies.

## Materials and methods

### Chemicals

Disodium-2,2-dimethyl-2-silapentane-5-sulphonate (DSS-d_6_) (CAT NO: sc-298862) and deuterium oxide 99.8% atomD (D_2_O) (CAT NO: sc-476928 A) were purchased from Santa Cruz. Di-potassium hydrogen phosphate (K_2_HPO_4_) (CAT NO: 1.05104.1000), 2-chloro-pyrimidine-5-carboxylic acid (CAT NO: CDS009961-50MG), LC/MS-grade water (CAT NO: 1.15333.2500), and sodium azide (NaN_3_) (CAT NO: 8.22335.0100) were purchased from Sigma Aldrich.

### Animals

In this study, the 5XFAD mouse model, which exhibits most of the phenotypic features of AD, was used. 5XFAD (B6SJLF1/J genetic background, RRID: IMSR_JAX:100012) mice were supplied from Jackson Laboratory (Bar Harbor, Maine, USA) by Acıbadem Mehmet Ali Aydınlar University, Experimental Animal Application and Research Center (ACU-DEHAM). All mice were housed in a controlled environment at a constant temperature (22 ± 2 °C) and maintained under a 12-h/12-h light/dark cycle. Mice were kept in regularly ventilated individually ventilated IVC (Individually Ventilated Cages). They have unlimited access to regular food and water with ad libitum.

In the experimental design of the study, 3, 6, and 9-month-old male 5xFAD (AD) and male wild-type (WT) mice (*n* = 3 per group) were used to determine age-related changes. All animal experiments were approved by the Acibadem Mehmet Ali Aydinlar University Ethics Committee for Experimental Research on Animals (ACU-HADYEK, Istanbul, Turkey, Approval ID: ACU-HADYEK2022/71) and performed in according to the institutional guide-lines.

### Collection of fecal samples and metabolite extraction

In the current study, different mice were used for each age group during the sample collection process. According to this, fecal samples were collected from 3, 6, and 9-month-old AD and WT mice in the cages, respectively. All collected samples were immediately transferred to 1.5 mL Eppendorf tubes and stored at -80 °C until the metabolite extraction.

For metabolite extraction, after thawing frozen fecal samples on ice, approximately 80 mg of wet sample was weighed into microcentrifuge tubes and dissolved in 750 µl of cold deionized water (4 °C). The solutions containing fecal samples were vortexed for 10 min and shaken at 200 rpm for 50 min at 4 °C. Then the mixtures were centrifuged at 15,000xg for 30 min at 4 °C. Supernatants transferred to 3 KDa cut-off centrifugal filter units (Amicon Microcon YM-3; Sigma-Aldrich, St. Louis, MO) were centrifuged at 13,000xg for 30 min at 4 °C. Prior to use, filters were washed 15 times with LC-grade water at 13.500xg for 10 min to remove glycerol from the filter. The resulting filtrates were transferred to the clean Eppendorf tubes (Miller et al. 2023). Additionally, the water content or dry-weight normalization of fecal samples was not measured.

### Sample preparation for NMR analysis

For the analysis of fecal metabolites in NMR spectroscopy, first, a standard buffer solution was prepared containing 1.75 M K_2_HPO_4_, 11.7 mM DSS-d_6_, and 5.84 mM 2-chloro-pyrimidine-5-carboxylic acid (phasing standard) in H_2_O, containing 0.04% NaN_3_ (w/v). Then, 570 µl of fecal extract solution was mixed with 60 µl of the buffer solution and 70 µl of D_2_O. The pH of the solution was measured and adjusted to 7.4 (Miller et al. 2023). 600 µl of final solutions were transferred to 5 mm SampleJet NMR tubes for analysis.

### 1H NMR-based metabolomics analysis

The NMR spectra analyses of fecal samples were performed by using a Bruker AVANCE Neo 600 MHz NMR spectrometer (Bruker Biospin, Rheinstetten, Germany) equipped with a 5-mm BBI Probe, Bruker SampleJet robotic system (regulated at a temperature of 7 °C) for sample cooling and Topspin 4.3.0 software (Bruker version 4.3.0). Nuclear Overhauser Enhancement Spectroscopy (NOESY) with “noesygppr1d” pulse sequence was used for the acquisition of 1D ^1^H NMR spectra, which were recorded at 300 K. In addition, the main parameters were set as follows: total scans; 32, data points; 64 K, spectral with; 20 ppm, dummy scans; 4, relaxation delay; 4 s, and total acquisition time; 4 min 4 s. The NMR spectra were referenced to the DSS-d_6_ peak at 0.0 ppm, and manually phase/baseline corrected using Topspin software (version 4.3.0, Bruker Biospin, Rheinstetten, Germany). Prior to NMR analysis, QC experiments were performed to obtain the best spectrum. The temperature-difference threshold at 300 K was 0.09. In water-suppression experiments, the threshold values for the reference signal’s line width and signal-to-noise ratio were 1 Hz and 300, respectively. All fecal samples were acquired on the same day in a single NMR run using identical acquisition parameters.

### NMR spectra profiling and NMR signal and metabolite validation

For samples analyzed by Chenomx spectra were processed with exponential line broadening such that the DSS peak width was 1 Hz. In all cases, following the zero and first order phase correction, manual baseline correction using *Whittaker Spline* function was applied. For manual profiling, fecal samples were quantified using the Chenomx NMR Suite version 10.1 (Chenomx, Inc. Alberta, Canada) using a combination of the software-provided 600 MHz compound library and an in-house compound library acquired at 600 MHz. Concentrations reported in this paper (µmol/L) are those in the NMR tube and have not been corrected for dilution with the buffer.

Spectral processing and metabolite profiling were not formally blinded. To verify precise alignment between experimental and reference signals, peak fitting was verified by manually examining spectral overlays in Chenomx. Targeted fitting against reference spectra was carried out in Chenomx for overlapping resonances, and signals that could not be consistently resolved were excluded from investigation. Chemical shift, multiplicity, and spectral fitting were used to assign metabolite identities with high confidence.

### Statistical analysis

The variances in metabolite concentrations between AD and control mice were statistically analyzed using Metaboanalyst 6.0. Data normalization was carried out using MetaboAnalyst 6.0’s normalization module, with the selected options of normalization by sum and Pareto scaling. An overview of the metabolic pattern changes between AD and WT mice was examined using principal component analysis (PCA). The threshold for statistical significance was *p*-value ≤ 0.05. The changes of potential metabolites linked to AD were identified by analyzing fold changes (FCs) with a cut-off of ≥ 1.2. Volcano plot analysis was used to determine important characteristics according to the biological and statistical relevance of metabolites. In volcano analysis, it was accepted a t-test *p*-value of ≤ 0.05 and a fold-change of ≥ 1.2 as threshold values for significant regulation of metabolites. As the supervised multivariate model, O-PLS-DA was performed to exploratively evaluate metabolic profile changes in fecal samples. In addition, permutation testing (100 permutations) and quality values R and Q of O-PLS-DA were measured on the Metaboanalyst 6.0 program. It should be noted that the permutation test performed in the Metaboanalyst 6.0 program is random. Therefore, *p*-values may vary. Furthermore, multivariate ROC curve analysis was performed. KEGG pathway enrichment analysis was used to map the potential pathways of metabolites. Box plots for the differential metabolites were created using “ggplot”package in R version 4.5.0 software.

## Results

### NMR-based profiling and metabolic pathways of untargeted fecal metabolites in 5XFAD and healthy mice

**Fig. 1:**
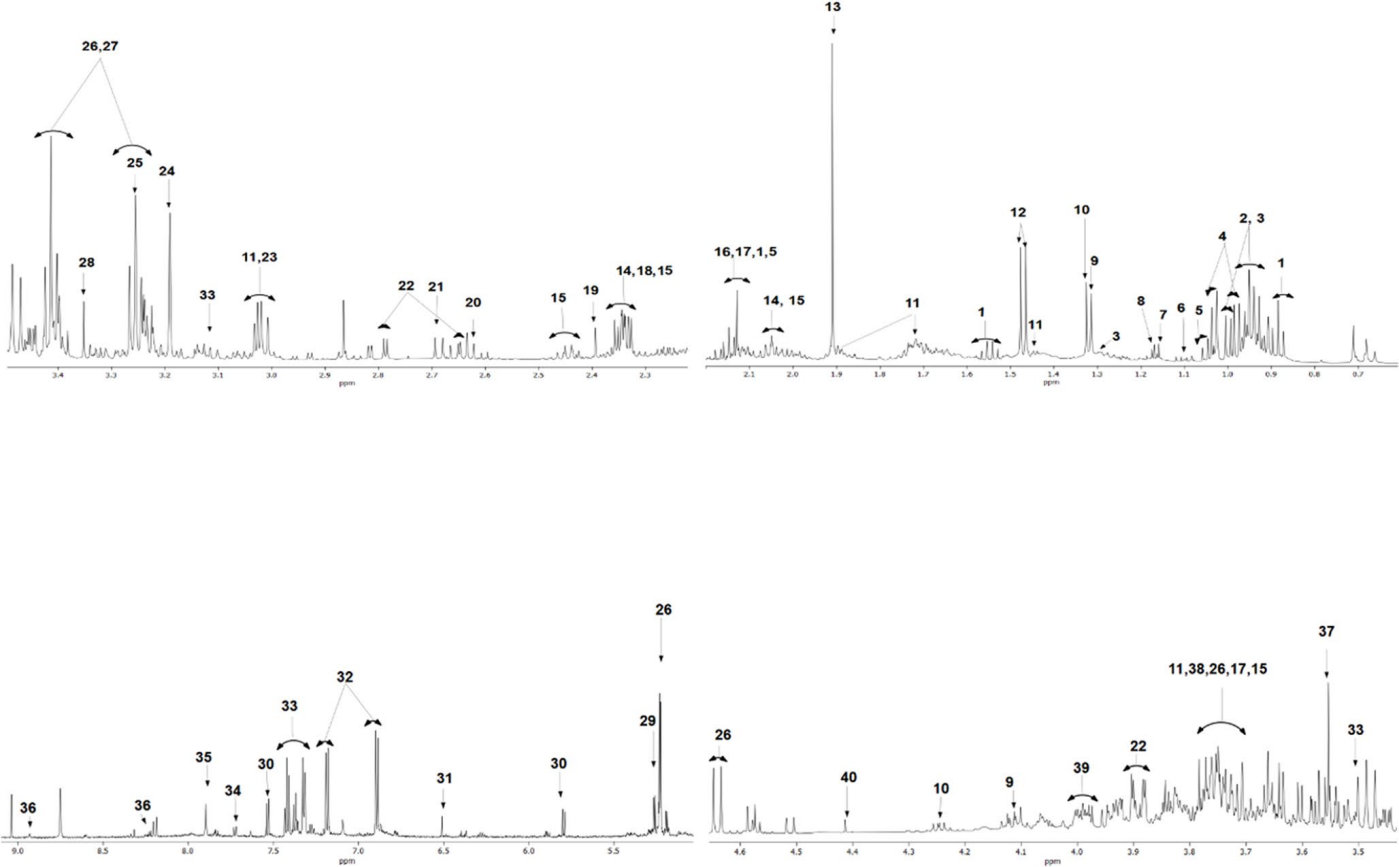
Regions of some identified metabolites in the 600 MHz 1 H-NMR spectrum of the pool mix of fecal samples used in this study (Spectral width: 20 ppm; 0 ppm: DSS-d_6_ peak; Total scans: 32; Temperature: 300 K). 1; butyrate, 2; leucine. 3; isoleucine, 4; valine, 5; propionate, 6; propylene glycol, 7; ethanol, 8; 3-Hydroxyisobutyrate, 9; lactate, 10; threonine, 11; lysine, 12; alanine, 13; acetate, 14; proline, 15; glutamate, 16; methionine, 17; glutamine, 18; pyruvate, 19; succinate, 20; methylamine, 21; malate, 22; aspartate, 23; creatinine, 24; choline, 25; trimethylamine N-oxide, 26; glucose, 27; taurine, 28; methanol, 29; galactose, 30; uracil, 31; fumarate, 32; tyrosine, 33; phenylalanine, 34; tryptophan, 35; xanthine, 36; nicotinate, 37; glycine, 38; fucose, 39; asparagine, 40; trigonelline

**Fig. 2:**
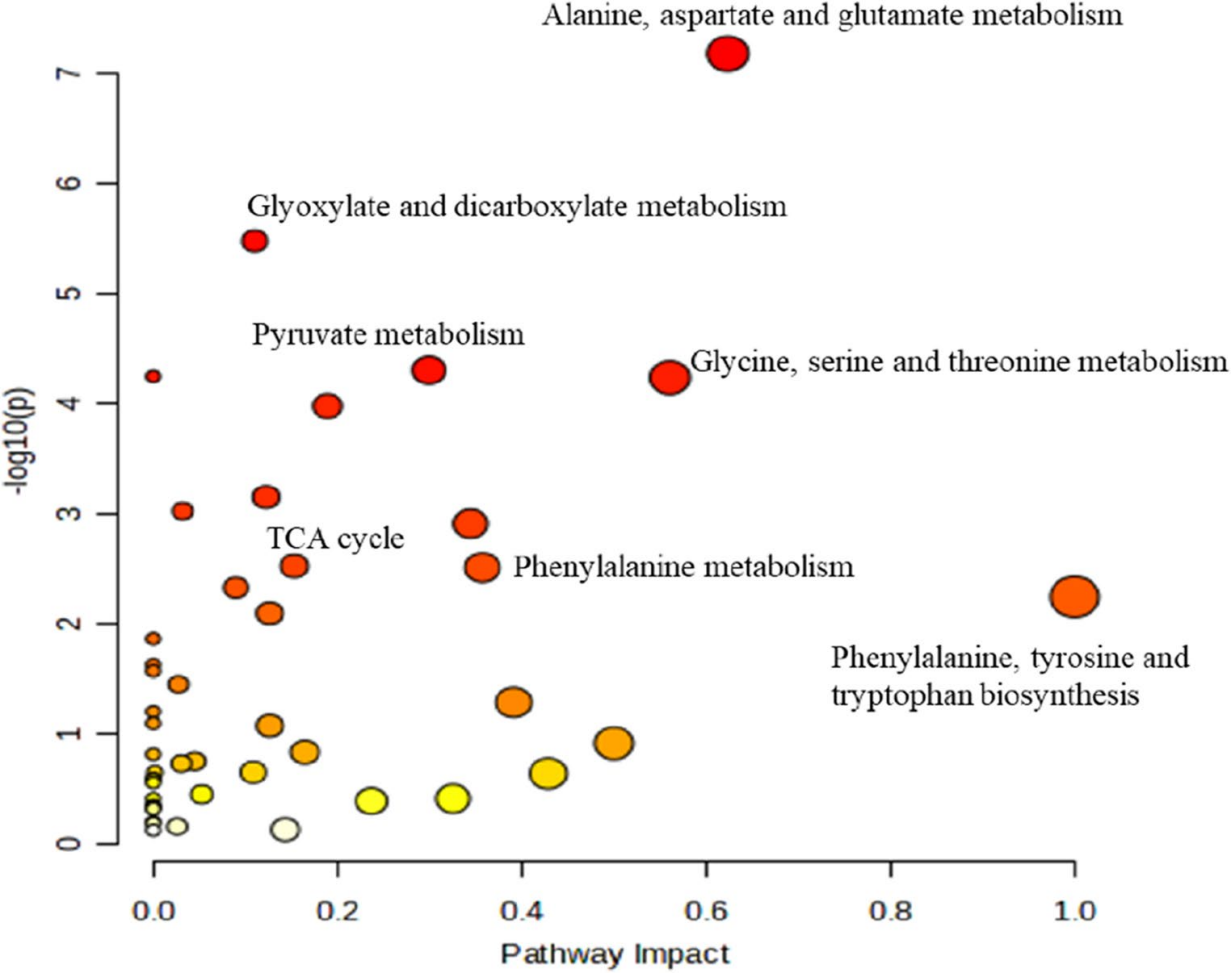
Pathway analysis diagram of fecal metabolites and some important metabolic pathways. Pathway analysis was used to determine the metabolic pathways associated with all fecal metabolites, and as the color diagram changes from red to light yellow, the *p*-value (≤ 0.05) increases

Fecal metabolic profiles in WT and 5XFAD mice were obtained using NMR spectroscopy. A representative NMR spectrum highlighting some of the metabolites identified in the 5XFAD mice is shown in Fig. 1. Regions of metabolites were assigned based on Chenomx NMR Suite version 10.1 (Chenomx, Inc. Alberta, Canada), the Human Metabolome Database (HMDB, http://www.hmdb.ca/), and the previous reported studies (Kostidis et al. 2017; Wang et al. 2020; Liu et al. 2021; Lee et al. 2023). As summarized in Table S1, a total of 67 metabolites were identified in all groups as a result of NMR-based metabolomic analysis of fecal samples. These metabolites were associated with 46 metabolic pathways, the majority of which are related to energy metabolism, amino acid metabolism, and nucleic acid metabolism. The full list of metabolite-associated pathways is shown in Fig. 2 and detailed in Table S2 where the color coding corresponds to that in the figure.

Among these pathways, the most significant enrichment was observed at alanine, aspartate, and glutamate metabolism, with its *p*-value of 6.7239 × 10^− 08^ and a pathway impact score of 0.6234. Moreover, the phenylalanine, tyrosine, and tryptophan biosynthesis mechanism has the highest pathway impact value as 1.0, and the *p*-value is 5.6854 × 10^− 03^.

**Fig. 3:**
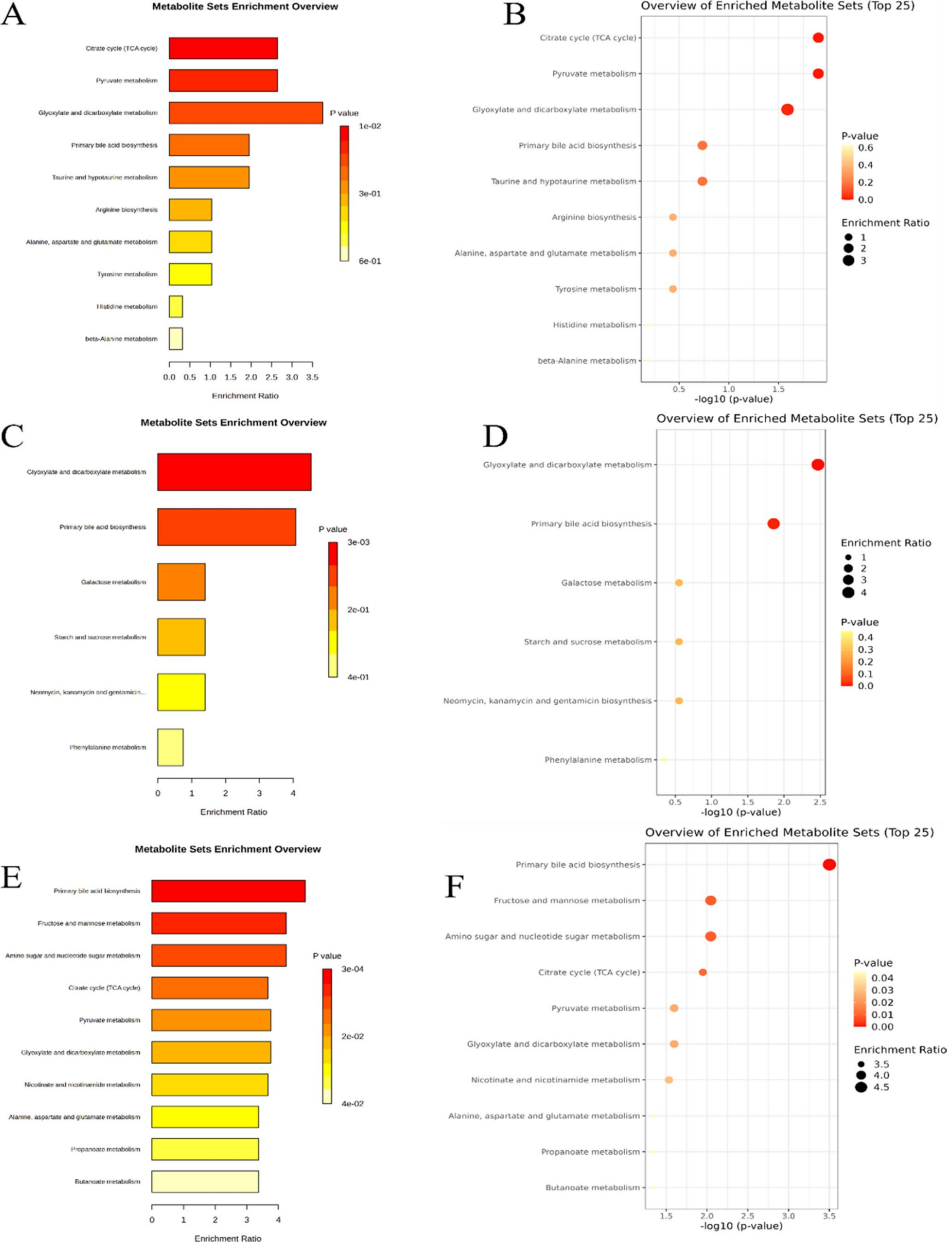
Results of metabolic pathways relating significant metabolites selected all age groups of 5XFAD mice. (**A**) enrichment ratio and (**B**) *p*-value for 3 M 5XFAD mice as a result of KEGG enrichment analysis. (**C**) enrichment ratio and (**D**) *p*-value for 6 M 5XFAD mice as a result of KEGG enrichment analysis. (**E**) enrichment ratio and (**F**) *p*-value for 9 M 5XFAD mice as a result of KEGG enrichment analysis. As the color diagram changes from red to light yellow, the *p*-value (≤ 0.05) increases

To assess differential metabolite levels between groups, volcano plot analysis with t-test (*p* ≤ 0.05) and fold change (≥ 1.2) was applied as a screening tool to 67 identified metabolites. The metabolic pathways associated with these differentially abundant metabolites at 3-month-old (3 M), 6-month-old (6 M), and 9-month-old (9 M) all mice were further analyzed using KEGG quantitative enrichment analysis (Fig. 3A-F). The significant metabolites between 3 M WT and 5XFAD mice were enriched in 10 metabolic pathways (Fig. 3A-B). The highest enrichment ratio (ER) was observed in glyoxylate and dicarboxylate metabolism (Fig. 3A), and the *p*-value was 0.02 (Fig. 3B). In this metabolic pathway, malate was significantly elevated in 3 M 5XFAD mice. Similarly, for citrate cycle and pyruvate metabolism, malate and fumarate were increased in 3 M 5XFAD mice (*p* = 0.01, ER = 2.65).

Additionally, the significant metabolites determined between all 6 M mice were enriched in 6 metabolic pathways (Fig. 3C-D). In the glyoxylate and dicarboxylate metabolism (*p* = 0.003, ER = 4.53), formic acid markedly decreased in 6 M 5XFAD mice. Cholate was significantly increased via the primary bile acid biosynthesis pathway (*p* = 0.01, ER:4.07) compared to the age-matched WT group. At 9 M, all significant metabolites were significantly enriched in association with all 10 metabolic pathways as shown in Fig. 3E-F.

The next section discusses age-specific alterations in fecal metabolite profiles in greater detail.

### Alteration of fecal metabolites in 3 M, 6 M, and 9 M WT and 5xFAD mice

**Fig. 4:**
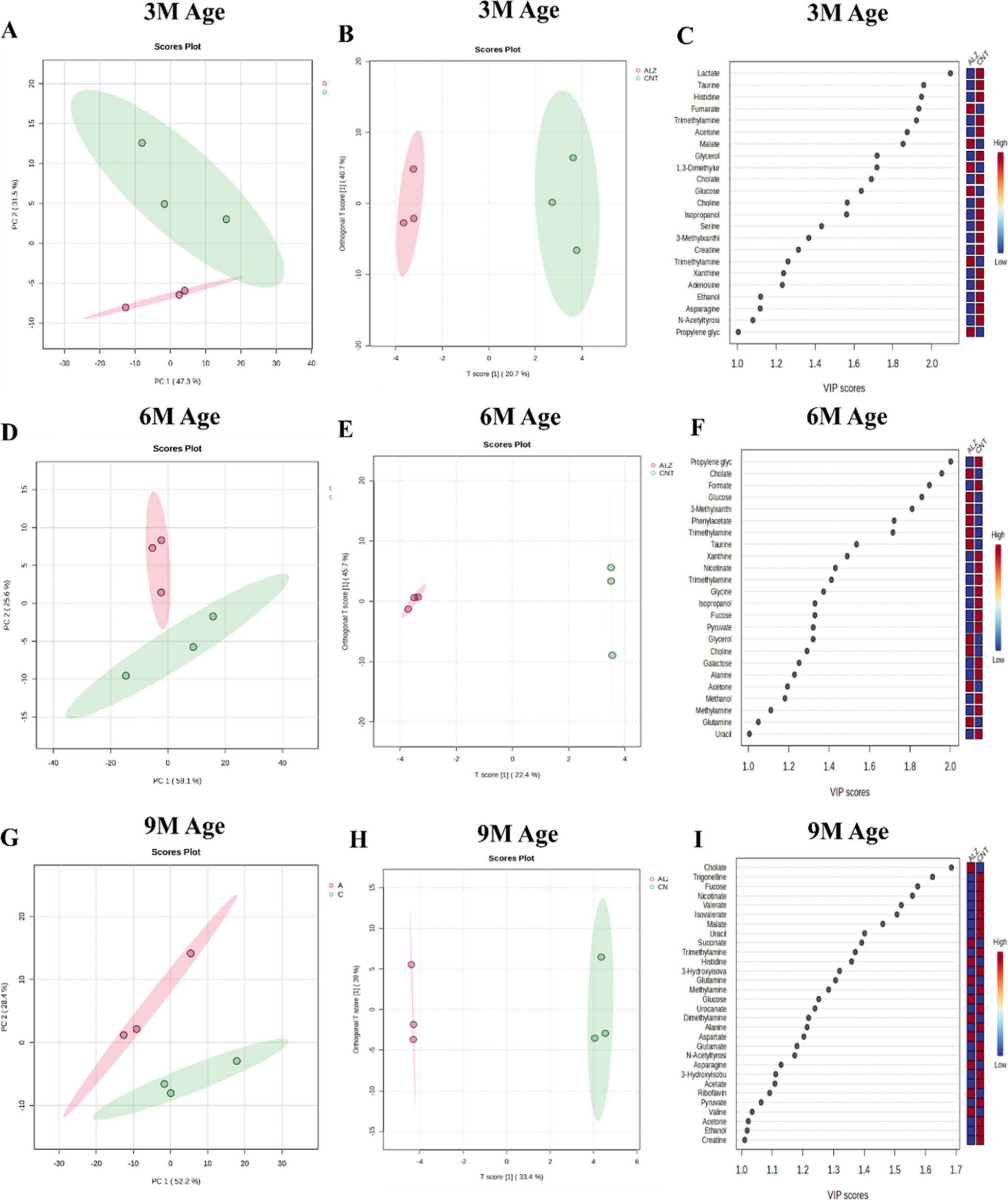
Separating 5XFAD and healthy mice in 3 M, 6 M and 9 M age groups using PCA score plot (**A**-**D**-**G**), two-component OPLS-DA score plot (**B**-**E**-**H**) and VIP scores (≥ 1.0) (**C**-**F**-**I**) according to fecal metabolite profiles. ALZ (Green in PCA and OPLS-DA, Red in VIP scores); 5XFAD mice, CNT (Pink in PCA and OPLS-DA, Blue in VIP scores); WT mice

PCA was performed to assess the changes in metabolic profile between groups. As shown in Fig. 4(A-I), 5XFAD and WT mice formed distinct clusters at 3 M, 6 M, and 9 M, indicating age-dependent alterations in fecal metabolites. Furthermore, orthogonal partial least squares discriminant analysis (O-PLS-DA) and VIP plot (VIP value > 1.0) was conducted solely for research and visualization purposes. As seen in Fig. 4C, F, I that, according to the T score from O-PLS-DA, the best VIP values were lactate (2.09) at 3 M, propylene glycol (2.00) at 6 M and cholate (1.68) at 9 M. However, these results provide a visual representation based on an analysis and do not represent a definitive conclusion depending on the sample size.

Additionally, the OPLS-DA analysis was subjected to model analysis and permutation. According to the results, in 3 M mice group, R²Y and Q² values of O-PLS-DA model were 0.94 and 0.71, respectively. These results demonstrated strong explanatory and predictive performance of model. Furthermore, there is a a reasonable difference between R²Y and Q², which indicates that overfitting is limited. According to permutation testing (100 permutations) performed, O-PLS-DA model validity has *p*-value > 0.05 for Q². According to this, it can be interpreted as exploratory due to permutation testing, although model shows biologically significant discrimination. Based on a 67-feature model, a multivariate ROC analysis (classification method: PLS-DA) produced an AUC of 0.90, suggesting very strong discriminatory ability. However, the results were interpreted exploratively because the broad confidence interval indicates poor resilience, perhaps as a result of sample size.

In 6 M mice group, The OPLS-DA model explained a significant amount of group variation (R^2^Y = 0.80) and had modest predictive ability (Q^2^ = 0.42). Nevertheless, statistical significance (*p* > 0.05 for Q^2^) was not confirmed by permutation testing (100 permutations), suggesting possible overfitting. This OPLS-DA model can be used for supportive and exploratory analysis because has not been validated in permutation testing although it performs moderately. With an AUC of 0.84, the multivariate ROC analysis based on a PLS-DA model showed good discriminatory performance. This suggests that the study groups are meaningfully separated by the chosen metabolite set. However, inadequate model stability is shown by the broad confidence interval (95% CI: 0–1), most likely as a result of sample size limitations.

In 9 M mice gorup, R²Y and Q² values for O-PLS-DA model were 0.89 and 0.68, respectively. Although these results shows good prediction accuracy and a significant amount of group variance, permutation test has *p* > 0.05 for Q². Because of the permutation test, this OPLS-DA model should be reported as supportive. With an apparent complete separation between groups, the multivariate ROC analysis (67-feature model and classification method: PLS-DA) produced an AUC of 1.0 with a 95% CI of 1–1. However, this conclusion is probably influenced by overfitting and evaluated cautiously due to the small sample size and high dimensionality of the data.

**Fig. 5:**
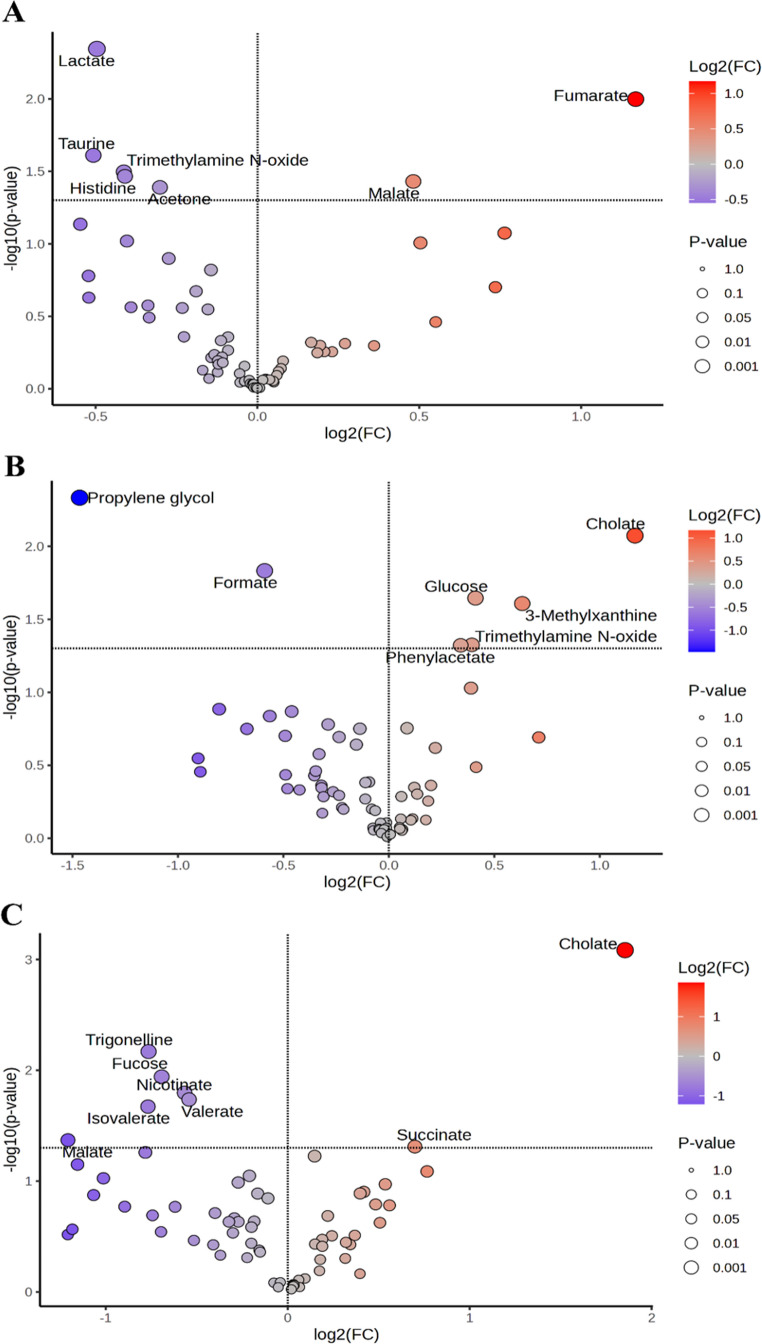
The significant metabolites in fecal samples for 3 M age (**A**), 6 M age (**B**), and 9 M age (**C**) according to the volcano plot analysis. The volcano plot analysis shows significant metabolites in fecal samples with *p*-value ≤ 0.05 and fold change ≥ 1.2. Dark blue-purple circles indicate down-regulated metabolites in 5XFAD mice, and orange circles indicate up-regulated metabolites in 5XFAD mice

**Fig. 6:**
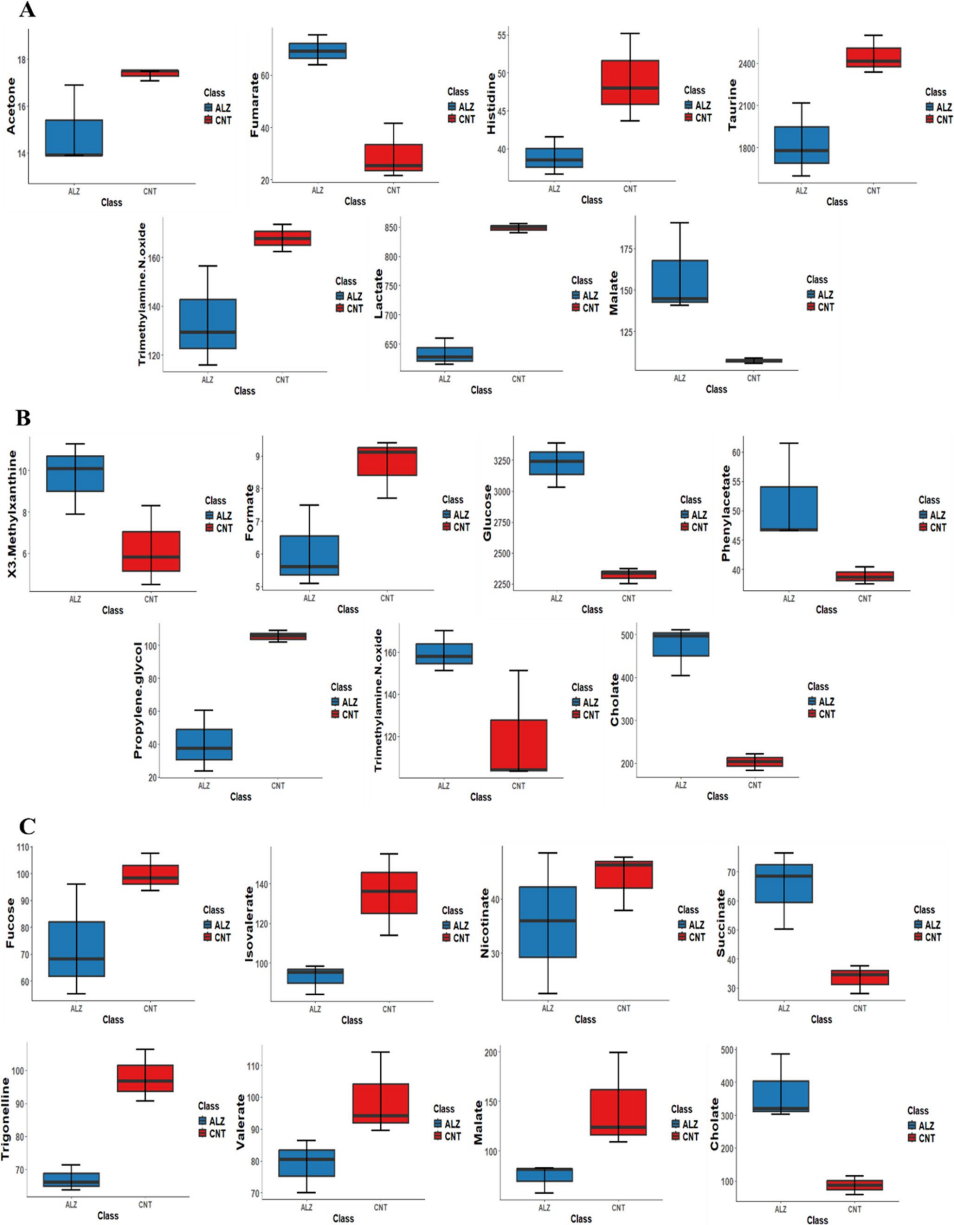
Box plots for the metabolites that changed significantly, identified through volcano plot analysis (p value ≤ 0.05 and fold change ≥ 1.2). These box plots display the upregulation or downregulation of significant metabolites between groups. ALZ (blue box); 5XFAD mice, CNT (red box); WT mice, x-axis; groups, y-axis; metabolite concentration (µmol/L)

According to the volcano plot analysis conducted for the exploratory identification of candidate biomarkers, Table 1; Fig. 5 show level changes in significant metabolites at different ages in AD mice compared to the control group. Furthermore, box plots relating to increasing and decreasing metabolites were shown in Fig. 6.

**Table 1:** The level changes of significant metabolites in different ages of 5XFAD mice compared to WT mice after the Volcano plot

	3 M 5XFAD Mice				6 M 5XFAD Mice				9 M 5XFAD Mice			
	Level	Fold Change	*p*-value (≤ 0.05)	Log2FC	Level	Fold Change	*p*-value (≤ 0.05)	Log2FC	Level	Fold Change	*p*-value (≤ 0.05)	Log2FC
3-Methylxanthine	-	-	-		+↑	1.55	0.02	0.63	-	-	-	-
Acetone	+↓	0.81	0.04	-0.30	-	-	-	-	-	-	-	-
Cholate	-	-	-	-	+↑	2.25	0.00	1.17	+↑	3.61	0.00	1.85
Formate	-	-	-	-	+↓	0.66	0.01	-0.59	-	-	-	-
Fucose	-	-	-	-	-	-	-	-	+↓	0.62	0.01	-0.69
Fumarate	+↑	2.25	0.01	1.17	-	-	-	-	-	-	-	-
Glucose	-	-	-		+↑	1.33	0.02	0.41	-	-	-	-
Histidine	+↓	0.75	0.03	-0.41	-	-	-	-	-	-	-	-
Isovalerate	-	-	-		-	-	-	-	+↓	0.59	0.02	-0.77
Lactate	+↓	0.71	0.00	-0.50	-	-	-	-	-	-	-	-
Malate	+↑	1.40	0.04	0.48	-	-	-	-	+↓	0.43	0.04	-1.21
Nicotinate	-	-	-	-	-	-	-	-	+↓	0.68	0.01	-0.57
Phenylacetate	-	-	-	-	+↑	1.27	0.05	0.34	-	-	-	-
Propylene glycol	-	-	-	-	+↓	0.36	0.00	-1.47	-	-	-	-
Succinate	-	-	-	-	-	-	-	-	+↑	1.62	0.05	0.70
Taurine	+↓	0.70	0.02	-0.51	-	-	-	-	-	-	-	-
Trigonelline	-	-	-	-	-	-	-	-	+↓	0.59	0.00	-0.76
Trimethylamine N-oxide	+↓	0.75	0.03	-0.41	+↑	1.31	0.05	0.39	-	-	-	-
Valerate	-	-	-	-	-	-	-	-	+↓	0.69	0.02	-0.54

At 3 M, acetone, histidine, lactate, taurine and TMAO were significantly decreased in 5XFAD mice, whereas fumarate and malate level were significantly increased (Table 1; Fig. 5A, and Fig. 6A). Moreover, in fecal samples of 6 M 5XFAD mice, the number of up-regulating metabolites, such as 3-Methylxanthine, glucose, phenylacetate and TMAO, increased (Table 1; Figs. 5B and 6B). As the disease progressed, the number and nature of altered metabolites expanded. For example, at 9 M 5XFAD mice, fucose, isovalerate, malate, nicotinate, trigonelline, and valerate decreased significantly, while cholate levels sharply increased with a fold change value of 3.61 (Table 1; Figs. 5C and 6C).

**Fig. 7:**
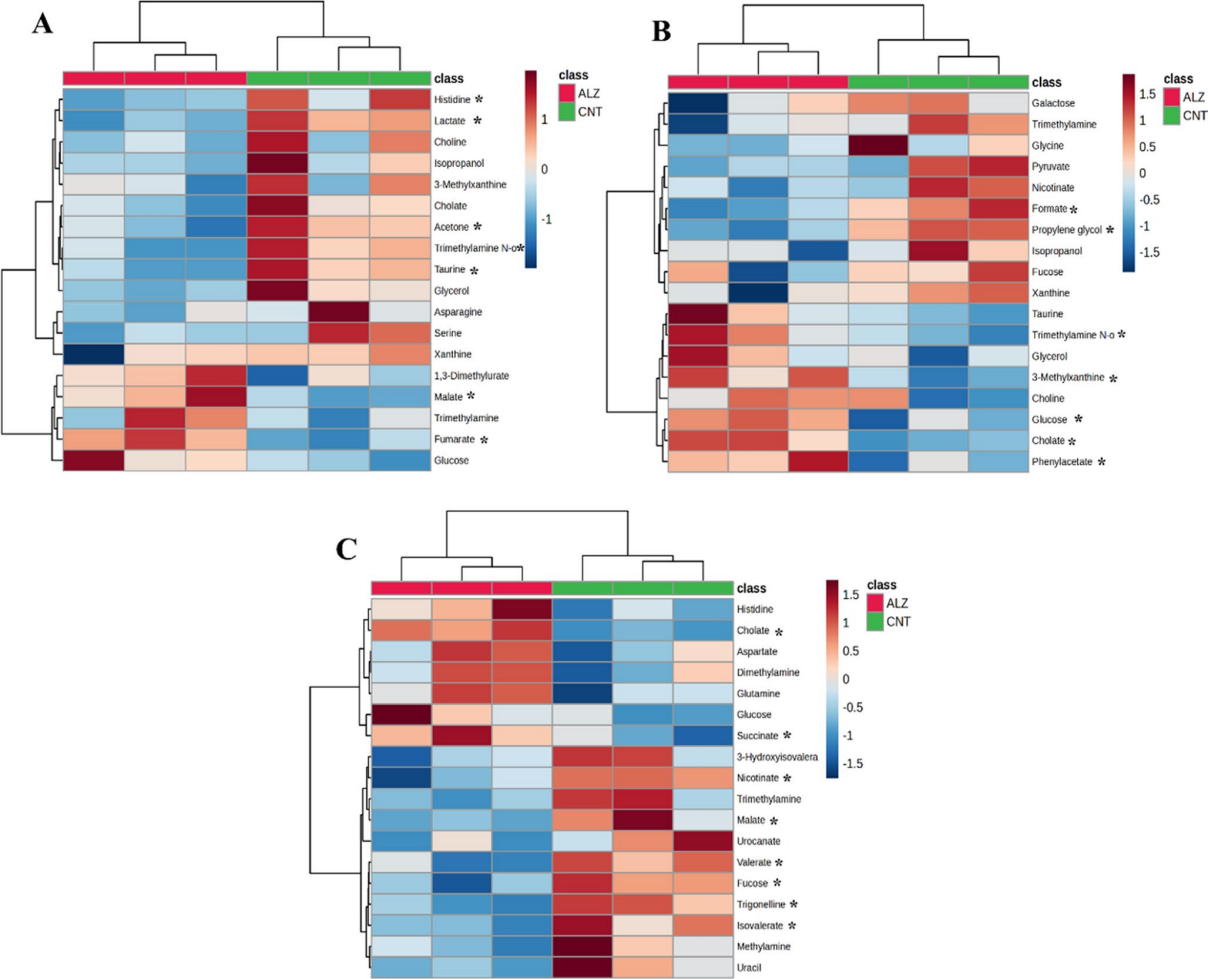
Heatmap diagram of the top 18 metabolites identified in 3 M (**A**), 6 M (**B**), and 9 M (**C**) mice. Heatmap analysis was conducted using t-test/ANOVA. Each colored cell on the heatmap indicates a concentration value for a metabolite. Rows and columns represent metabolites and samples, respectively. The symbol “*” defines the significant metabolites according to volcano plot analysis (p-value ≤ 0.05 and fold change ≥ 1.2). ALZ (Pink); 5XFAD mice, CNT (Green); WT mice, Blue squares; down-regulated metabolites, Orange squares; up-regulated metabolites

As shown in Table 1, age-dependent alterations in metabolite profiles were evident across the 5XFAD groups. The number of down-regulated metabolites was higher at 3 M and 9 M, whereas 6 M mice showed an increase in up-regulated metabolites. Heatmaps based on t-test and ANOVA analyses were performed to visualize metabolite concentrations across groups. The top 18 differentiating metabolites, regardless of statistical significance, are shown in Fig. 7. Analysis of metabolite levels and diversity across age groups revealed that some metabolites were shared among multiple ages, while others can be associated with specific age groups. For instance, TMAO was altered both in 3 M and 6 M; it decreased in 3 M and significantly increased in 6 M mice. Malate followed the opposite trend; elevated in 3 M but reduced in 6 M. In contrast cholate was consistantly elevated in both 6 M and 9 M mice.

Additionally, FDR correction for significant metabolites was performed to remove false positive effect. However, after the FDR correction, no statistically significant metabolites remained in all the age groups. This situation may be due to the small sample size (*n* = 3). Because, as the sample size increases, the *p*-value decreases and the statistical power increases. In addition, since the FDR correction depends on the number of hypotheses tested, identifying significant metabolites after FDR correction becomes challenging in small samples. In this sense, the present study aimed to investigate potential candidate metabolites for AD-related changes only in fecal samples.

Furthermore, to visualize the age-stratified cross-sectional comparison of metabolites in three different-aged AD and WT mice, PCA and Sparse PLS-DA (sPLS-DA) were performed by using determined metabolites (Figure S1-A and Figure S1-B). According to the PCA, in the AD group containing 3 M, 6 M and 9 M mice, As AD progresses, a change in the metabolite profile is observed (Figure S1-A). The 3 M time point, in particular, tends to exhibit a significantly different metabolic profile compared to the later stages of AD (6 M and 9 M). However, overlapping metabolites are observed in the 6 M and 9 M time points, when neurodegeneration becomes more pronounced. Furthermore, a shift toward PC2 was observed in the fecal metabolite profile of 9 M mice. In the sPLS-DA analysis of AD group, the separation is more clearly visible according to metabolites best fitted the model. While the 3 M group was completely separated from the other time points, the 6 M group exhibited a transitional trend in fecal metabolite profile between the 3 M and 9 M groups. The 9 M group showed a profile that progressed in a different direction from the other groups, with clearer signs of advanced AD.

In addition, when The PCA graph for the healty group was evaluated, certain metabolic overlaps are evident throughout all stages of the aging process (Figure S1-C). However, the sPLS-DA plot, which analyzes the groups more clearly, demonstrates that the metabolic profile can clearly change with aging (Figure S1-D). In this sense, when comparing the age-stratified cross-sectional metabolic profile changes in the AD and healthy groups, both groups show different trends in metabolite changes due to aging. While a slight metabolic overlap with aging was observed in the 6 M and 9 M groups in AD, the metabolic profiles differed significantly across all age groups in healthy mice.

## Discussion

The pathophysiology of AD, a neurodegenerative disease marked by cognitive decline, remain highly complex. The number of dementia sufferers is rising, associated with the population ages. On the other hand, current pharmacological treatments for AD are limited in their efficacy and often associated with adverse effects (Fu et al. 2023). Recent studies indicate the significant role of the gut microbiota in the development and progression of AD (Liang et al. 2024). Researchers are indeed exploring the gut microbiota-brain axis to develop effective diagnostic and therapeutic strategies. Microbial metabolites play a crucial role in mediating the bidirectional communication between the gut microbiome and the brain, influencing various physiological processes and disease states. Consistent with previous studies, our exploratory findings in this pilot study showed that the fecal metabolic profile can be changed due to aging in AD. Several metabolites identified in this study have been previously associated with AD, including taurine (Huf et al. 2023; Ahmed et al. 2024), lactate (Ling et al. 2021; Khedr et al. 2022), TMAO (Vogt et al. 2018; Mudimela et al. 2022), trigonelline (Farid et al. 2020; Feng et al. 2024), formate (Bridgman et al. 2017; Wu et al. 2021), valerate (Senarath et al. 2024), 3-methylxanthine (Paglia et al. 2016; Zhang et al. 2023; Qu et al. 2024), acetone (Henderson 2010; Shippy et al. 2024), cholate (Shao et al. 2020; Fu et al. 2023), fucose (Lucente et al. 2022), glucose (Butterfield and Halliwell 2019), histidine (Song et al. 2018; Nielsen et al. 2021), isovalerate (Chen et al. 2024; Kuehn et al. 2024), malate (Wang et al. 2014; Milos et al. 2023), nicotinate (Fricker et al. 2018), phenylacetate (Huang et al. 2023; Ren et al. 2024), succinate (Jaramillo-Jimenez et al. 2023; Al-abbas and Shaer 2024).

SCFAs are produced via bacterial fermentation of indigestible carbohydrates by the gut microbiota (Bridgman et al. 2017; Swer et al. 2023). They have critical roles on mucus formation, inflammation response, and intestinal barrier maintenance (Carabotti et al. 2015). The most abundant SCFAs are acetate, butyrate, and propionate, along with other SCFAs such as valerate, isovalerate, and isobutyrate, which are produced in smaller amounts. SCFAs can influence with the gut-brain axis by binding to receptors expressed on various cells within this communication network, including enteroendocrine cells, immune cells, and neurons (Nøhr et al. 2015; Caspani and Swann 2019). They can also cross the blood-brain barrier (BBB) and interact with microglia (Wenzel et al. 2020).

In our study, SCFAs such as isovalerate, valerate, and formate, lactate and succinate can be suggested as significant fecal candidate discriminant metabolites. It is reported in the literature that valerate is positively associated with amyloid beta accumulation (Senarath et al. 2024) and endothelial disruption (Marizzoni et al. 2020). On contrary, another study reported that, isovalerate and valerate were found to have a negative correlation in abstraction from specific cognitive domains (Chen et al. 2024). In line with these findings, we observe that, valerate and isovalerate were significantly decreased in 9 M 5XFAD mice. In addition, research has highlighted the potential therapeutic benefits of certain SCFAs, such as valeric acid, butyric acid, and propionic acid, in disrupting protein-protein interactions involved in the aggregation of Aβ peptides, a key feature of AD pathology. These findings suggest a possible role for these compounds in modulating disease progression. In particular, valeric acid has been shown to most strongly inhibitor for the conversion of Aβ1–40 and Aβ1–42 into Aβ fibrils by protein-protein interaction by electron microscopy based examination (Ho et al. 2017). In this context, altered SCFA levels resulting from gut microbiota dysbiosis may be associated with AD. Furthermore, in the current study, formate levels also decreased significantly in 6 M 5XFAD mice, similarly to isovalerate and valerate. In a way that may interpret these results, Wu et al. (2021) observed that formate decreased significantly in the different AD processes (amnestic mild cognitive impairment (aMCI) and dementia (AD) in human fecal samples compared to the healthy group. Additionally, it was determined that isovalerate decreased significantly in aMCI and AD processes, while valerate decreased significantly only in AD.

Previous studies reported that dysregulated bile acid metabolism is closely tied to AD, with changes in gut microbiota composition and metabolic activity along the gut-brain and gut-liver-brain axes playing a pivotal role in AD’s pathophysiological mechanisms. Cholesterol is converted in the liver into cholic acid and chenodeoxycholic acid, the primary bile acids, which are then transported to the intestine for further metabolism by the gut microbiota (MahmoudianDehkordi et al. 2019). However, alterations in gut microbial composition may disrupt primary bile acid metabolism, suggesting a strong association between abnormal intestinal bile acid processing and AD (Shao et al. 2020; Fu et al. 2023). This underscores the intricate relationships between the gut microbiome, metabolic processes, and brain function in AD. Fu et al. (2023) reported changes in cholic acid levels due to microbial composition in fecal samples. For instance, the genera *Bacteroides*,* Coprococcus*, and *Paraprevotella* were negatively associated with cholate levels, and their increased abundance was suggested to exert neuroprotective effects. Shao et al. (2020) found that serum levels of cholic acid and other bile acids increased significantly with disease severity, suggesting a potential correlation between bile acid metabolism and AD progression. Consistently, in this pilot study, cholate levels tended to increase in fold change, rising from 2.25 to 3.61, as AD severity progressed from 6 M 5XFAD mice to 9 M 5XFAD mice.

Lactate is the prevalent short-chain hydroxy– fatty acid in the intestinal lumen and can be converted to other SCFAs by lactate-fermenting bacteria (Iraporda et al. 2015). Lactate level is positively correlated with AD due to the presence of lactate-fermenting microorganisms in AD (Ling et al. 2021; Khedr et al. 2022). However, we observed a decrease in lactate levels in 3 M 5XFAD mice and no significant change was observed in the later stages of AD. Contrary to the literature but similar to ours, Saji et al. (2020) reported that higher fecal lactic acid levels were associated with lower dementia risk, implying potential cognitive benefits from lactic acid bacteria.

Another candidate metabolite identified in this study is malate, which shows an age-dependent pattern in 5XFAD mice—elevated in 3 M compared to age-matched WT controls but tended to decrease at 9 M with increasing age. As a key intermediate component in the TCA cycle, its concentration can reflect alterations in this metabolic pathway. Prior studies have reported a similar decline in malate levels from healthy controls to MCI and AD patients (Wang et al. 2014; Milos et al. 2023), suggesting impaired energy homeostasis due to dysregulation of both glycolysis and TCA cycle in AD.

Phenylacetate is a product of phenylalanine in the amino acid metabolic pathway. Abnormalities in phenylalanine metabolism can lead to the accumulation of phenylacetate and phenylethylamine, which may impair neuronal function and cognitive ability, thereby contributing to the onset and progression of AD (Huang et al. 2023; Ren et al. 2024). It has also been reported that phenylacetate levels are significantly increased in AD model organisms due to impaired amino acid metabolism (Ren et al. 2024). Consistent with these findings, candidate phenylacetate metabolite had an increase in 6 M 5XFAD mice in our study. Furthermore, the phenylalanine, tyrosine, and tryptophan biosynthesis pathway showed the highest pathway impact value in the enrichment analysis. These results suggest that there may be potential changes in both metabolite levels and related metabolic pathways.

Among the other metabolites associated with neurodegenerative disorders, nicotinate is a metabolite linked to the nicotinate/nicotinamide pathway in energy metabolism. Nicotinamide, the primary absorbable form of vitamin B3 from animal-based diets, is converted into nicotinate by bacterial nicotinamidases in intestinal lumen (Braidy et al. 2019). There is evidence that nicotinamide and nicotinate have neuroprotective effects such as regulating cholesterol metabolism, improving neuronal mitochondrial function, reducing oxidative stress in AD and the other neurodegenerative disorders (Fricker et al. 2018). In the line with this, in the current study, levels of nicotinate were less abundant in fecal samples of 9 M 5XFAD mice. These findings are compatible with the previous studies and suggest that the neuroprotective effects of nicotinate may be diminished due to its decreased abundance.

Succinate, another TCA intermediate, plays a critical role in the TCA cycle and contributes to ATP production through oxidative phosphorylation. With aging, mitochondrial functions—particularly the efficiency of the TCA cycle—decline, leading to impaired energy production. Dysregulated succinate levels have been reported in the brains of AD patients, which are believed to contribute to mitochondrial dysfunction and oxidative stress (Al-abbas and Shaer 2024). Succinate accumulation can lead to mitochondrial ROS production, oxidative stress, and neuronal damage (Swerdlow et al. 2010). In the present study, exploratory analysis results showed a significant increase in succinate levels in 9-month-old 5XFAD mice. These findings highlight the complexity of the relationship between gut microbiota, succinate metabolism, and AD pathophysiology, underscoring the need for further investigation.

The metabolites, such as fucose and glucose, generated through energy metabolism have distinct effects on brain functions. For example, L-fucose is a novel signaling molecule capable of regulating synaptic neurotransmission via a metabolic signaling mechanism, and in the brain, fucosylation has been reported to influence processes involved in learning and memory, such as synapse formation, neurite outgrowth and migration, and long-term potentiation. 5XFAD mouse brains showed signs of fucose hypometabolism with impaired L-fucose signaling (Lucente et al. 2022). Consistent with this mechanism, the fucose levels in our study may decrease in 9 M 5XFAD mice. Lucente et al. (2022) also observed that dietary L-fucose supplement alleviated synaptic and behavioral deficits of 5xFAD mice.

Similar to fucose metabolism, the brain primarily needs glucose for energy, but glucose metabolism is drastically reduced in AD and MCI. Likely due to oxidative damage affecting enzymes involved in ATP production, glycolysis, and the TCA (Butterfield and Halliwell 2019). However, in the current study, 6 M 5XFAD mice showed a considerable increase in glucose levels in fecal samples. Additionally, glucose levels in 3 M 5XFAD mice were higher than in age-matched WT mice, although this difference was not statistically significant. In contrast, the concentration of glucose in 9 M 5XFAD mice did not change compared to 6 M 5XFAD mice, and its concentration was constant in all age groups of WT mice. The stability of glucose metabolism in the control group, compared with the variability observed in the Alzheimer’s group suggests age-related impairment in glucose metabolism in 5XFAD mice. These metabolic alterations serve as important secondary evidence of AD progression in this model. The observed metabolic dysregulation further supports the link between metabolic changes and increasing AD severity in these animal models. However, the results obtained here are exploratory and require further verification.

Histidine, which was found to be statistically significant in this study, has been demonstrated to have various neuroprotective properties regarding cerebral hypoperfusion, promoting neurogenesis, and the integrity of the blood-brain barrier after disruption (Song et al. 2018; Nielsen et al. 2021). Histidine levels were decreased in 5XFAD mice in the pilot study, consistent with the findings of Nielsen et al. (2021), likely due to impaired histidine metabolism. Moreover, lower acetone levels in 3 M 5XFAD mice were in line with high glucose concentration. In AD, glucose hypometabolism limits glucose utilization, leading to the increased use of alternative energy sources such as acetone and other ketone bodies (acetoacetate and β-hydroxybutyrate) serve as an alternative energy (Henderson 2010; Shippy et al. 2024). However, in our study, the higher glucose concentration in 3 M 5XFAD mice likely reduced the reliance on ketone bodies, resulting in decreased acetone levels. Additionally, 3-methylxanthine, a derivative of xanthine, has been reported at elevated levels in serum or brain tissues of individuals with AD disease and cognitive dysfunction according to reports such as Zhang et al. (2023), Paglia et al. (2016), Qu et al. (2024), similar to 3-methylxanthine levels in fecal samples of the present study. Although this increase was significant only in 6 M 5XFAD mice, also a slight age-dependent upregulation in 3-methylxanthine was observed from 3 M to 9 M 5XFAD mice.

Trigonelline, a methylation product of niacin (vitamin B3), is a pyridine alkaloid (Farid et al. 2020). Trigonelline has been reported improving neuronal loss caused by oxidative stress, cognitive decline, brain lesions, depression, stroke (Fahanik-Babaei et al. 2019; Liang et al. 2023; Feng et al. 2024). In addition, trigonelline has been shown to alleviate cognitive impairment by repairing brain damage, increasing the activity of the antioxidant system in mice, and controlling the activity of AChE and ChAT to keep the cholinergic system in balance (Feng et al. 2024). On the other hand, in the present study, exploratory analyses revealed a tendency for trigonellin to decrease in 9 M 5XFAD mice. Specifically, this decrease persisted from 3 M to 9 M 5XFAD mice. Accordingly, changes in trigonellin levels may be associated with the progression of AD.

TMAO is a metabolite produced as a result of the fermentation of dietary choline, phosphatidylcholine, carnitine, and betaine by anaerobic microorganisms in gut microbiota (Day-Walsh et al. 2021). Most studies showed that the CSF and plasma samples and brain tissues obtained from AD patients or animal models of AD had higher levels of TMAO (Vogt et al. 2018). It was determined that these increased TMAO levels lead to oxidative stress in hippocampus slices by upregulating reactive oxygen types (free radicals), hydrogen peroxide, lipid peroxidation, and other chemicals (Mudimela et al. 2022). Studies using CSF have shown that TMAO can cross the blood-brain barrier (Vogt et al. 2018; Enko et al. 2020), and increased levels of TMAO have been linked to enhanced neuroinflammation, astrocyte activation, and upregulation of pro-inflammatory mediators, all of which contribute to neuroinflammatory responses (Mudimela et al. 2022). Additionally, in our current study, a decrease in candidate TMAO metabolite levels was observed in 3-month-old 5XFAD mice compared to controls, while an increase in these levels was seen in 6-month-old AD mice compared to controls, demonstrating consistency with the information reported in the literature.

Current findings in the pilot study suggest a possible decrease in taurine levels in 3 M 5XFAD mice. In 6 M 5XFAD mice, a non-significant increase was observed compared to age-matched WT mice. In 9 M 5XFAD mice, where the AD phenotype is more pronounced, taurine levels may be lower compared to the control group. Similarly, Fujii et al. (2019) found that fecal microbiota transplantation (FMT) from AD patients resulted in decreased levels of taurine and other metabolites in mice, compared to those receiving microbiota from healthy individuals. Taurine, an abundant amino acid in living organisms, can play a role in various physiological processes, including neuroprotection, neuromodulation, and osmoregulation, among others (Kumari et al. 2013; Wang et al. 2021; Yildirim et al. 2023). Furthermore, recent studies have shown that taurine supplementation improves cognition in AD (Huf et al. 2023; Ahmed et al. 2024). In that regard, decreasing taurine in 5XFAD mice in this study can be associated with disease progression.

The effect of propylene glycol on the gut-brain axis is complicated. Propylene glycol, also known as 1,2-propanediol, is frequently used as a solvent, plasticizer, humectant, bacteriostat, and fungistat in pharmaceuticals, cosmetics, and food. Especially, in semimoist pet food, propylene glycol is frequently utilized as a cost-effective source of metabolizable energy (Plumlee 2004). Furthermore, it was shown that in the hippocampal region of male rat, propylene glycol and amyloid beta1-40 toxin cause excessive neurodegeneration by increasing lipid peroxidation and reducing superoxide dismutase in the rat brain (Salari and Bagheri 2024). This chemical, through which enters the body different routes, can be metabolized in the liver to lactate, pyruvate, and acetate (Lim et al. 2014). In addition, the presence of this chemical has been shown in fecal samples (Chow et al. 2014; HMDB 2023). In the current study we observed a significant decrease in propylene glycol levels in the fecal samples of 6 M 5XFAD mice compared to controls. However, it remains unclear whether it is associated with AD pathology or, nutrition, or metabolism of AD mice, warranting further research to elucidate the underlying mechanisms.

This study presents preliminary findings consistent with trends reported in the current literature regarding potential metabolites associated with AD. The majority of metabolites previously reported in the literature exhibited gradual alterations in the AD mouse model in parallel with disease progression over time. These changes demonstrated a notable correlation with established data, suggesting that these metabolites may play a critical role in the pathophysiology of AD. Furthermore, AD exhibits unique pathophysiological characteristics impacted by biological sex, with women being disproportionately afflicted due to sex-specific hormonal, epigenetic, and genetic variables (Saha and Sisodia 2024). In addition, female animals in biomedical research are traditionally mostly excluded from research studies due to the perceived additional complexity caused by the estrous cycle (Chari et al. 2020). However, male reproductive hormones are relatively stable throughout much of the young adult lifespan, and therefore males tend to exhibit less variability in behavioral and physiological assessments (Mennenga and Bimonte-Nelson 2015). Therefore, because the current study has focused on the effect of AD on the fecal metabolomics, male mice were preferred across all age groups to provide the homogenization and stabilization of the changes in the fecal metabolite profile associated with AD. In addition, although the water content in fecal samples may contribute to variability in absolute metabolite levels (Erben et al. 2021; Sangaraju et al. 2024), similar to the current study, most fecal metabolomics studies in the literature (Fujii et al. 2019; Dunham et al. 2022; Zheng et al. 2022; Ubeda et al. 2023; Zhao et al. 2025) do not include water content of collected fecal samples. In the current study, every fecal sample was collected from laboratory mice housed in controlled environments with standardized food, a light-dark cycle, and health and husbandry practices. Compared with human samples, these controlled conditions are expected to reduce inter-individual variability in fecal composition and water content. Additionally, in the present study, sample collection and metabolite extraction were performed according to a specific procedure. However, residual variability cannot be ruled out because fecal water content was not explicitly quantified. Therefore, since controlled individuals and standard procedures were used, the variability in absolute metabolite concentrations due to the water content of the fecal sample was minimized. Furthermore, since this preliminary study was conducted with a small sample size (*n* = 3), the identified fecal metabolites are considered exploratory. Research with a larger sample size is needed to clarify the consistency and significance of the statistical analyses performed.

When evaluating the changes in candidate fecal metabolites observed in our pilot study, it was found that most metabolites showed a decreasing trend in 3- and 9-month-old 5XFAD mice. In contrast, a majority of metabolites were increased in 6-month-old 5XFAD mice. As demonstrated in our previous study (Uras et al. 2023), the protein expression profiles of neonatal AD mice resemble those of post-mortem human brain samples from AD patients. However, this similarity diminishes over time and re-emerges in aged mice. In light of these findings, the present study serves as a pioneering exploratory investigation suggesting that the metabolite trends observed in the early developmental stages of 5XFAD mice resemble the fecal metabolite patterns associated with AD during aging, similar to the proteomic patterns shown in the previous study. Furthermore, significant differences were observed between metabolite profiles identified at different age groups. Therefore, more comprehensive studies are needed to clarify the causal relationships underlying changes in metabolite profiles. Metabolomics studies do not provide clear information about the bacterial species in the gut-microbiota axis and the specific metabolites they produce. Therefore, the combined application of several different omics technologies is a powerful approach to obtain detailed and precise results about the axis (Daliri et al. 2021). It is hoped that these metabolites will have better pharmacological effects on hosts and can be developed as new drugs for the diagnosis and treatment of AD disease, thanks to clear and detailed results that support each other with multi-omics approaches. In particular, new concepts can be developed in personalized medicine, such as the creation of individual nutritional approaches for neurodegenerative disorders by utilizing an individual’s unique gut microbiota (Chunduri et al. 2022). In the current study, identifying pathways related to disease progression using fecal metabolites is not entirely sufficient to establish a cause-and-effect relationship in the AD process. While our findings, as reported in the literature, exploratively demonstrate links between fecal metabolite profiles and AD pathology, further experimental planning is needed to determine whether these metabolite changes trigger neurodegeneration or occur as a consequence of the disease. Interventions such as FMT or metabolite supplementation or consumption could provide important information regarding gut microbiota regulation, neuroinflammatory responses, and Aß or tau accumulation. Supporting these studies with multiomics approaches will help clarify the biological significance of the pathways identified in this study.

## Conclusion

In this pilot study, a total of 67 potential metabolites were identified in fecal samples of 5XFAD and WT mice at different time points. Among these metabolites, 19 showed significant upregulation or downregulation in 5XFAD mice compared to WT mice during the aging process. These candidate metabolites included fumarate and malate in 3-month-old 5XFAD mice; 3-methylxanthine, glucose, cholate, phenylacetate, and TMAO in 6-month-old 5XFAD mice; and cholate and succinate in 9-month-old 5XFAD mice. These metabolites were associated with various metabolic pathways such as the TCA cycle (fumarate, malate, succinate), pyruvate metabolism, glyoxylate and dicarboxylate metabolism (fumarate, malate), primary bile acid biosynthesis (cholate), galactose metabolism and starch-sucrose metabolism (glucose), phenylalanine metabolism (phenylacetate), alanine, aspartate and glutamate metabolism, propanoate and butanoate metabolism, purine metabolism (3-methylxanthine), and trimethylamine metabolism (TMAO). These exploratory findings aim to contribute to the literature by enhancing our understanding of fecal metabolome alterations and their relationship with the AD model 5XFAD mice. 

## Supplementary Information

Below is the link to the electronic supplementary material.

Supplementary Material 1 (DOCX 17.8 KB)

Supplementary Material 2 (DOCX 337 KB)

Supplementary Material 3 (DOCX 3.02 KB)

Supplementary Material 4 (DOCX 3.05 KB)

Supplementary Material 5 (DOCX 3.01 KB)

Supplementary Material 6 (DOCX 368 KB)

Supplementary Material 7 (DOCX 21.2 MB)

## Data Availability

The data supporting this article have been included as part of the Supplementary Information.
