# Supplementary figures and images for: A perspective for alzheimer disease from gut microbiota-associated NMR-based fecal metabolomics: a study with 5XFAD mice
Source: Metab Brain Dis. 2026 Apr 24;41(1):91. doi: 10.1007/s11011-026-01848-2 (PMC13109219; doi:10.1007/s11011-026-01848-2)

**Graphical Abstract**

**
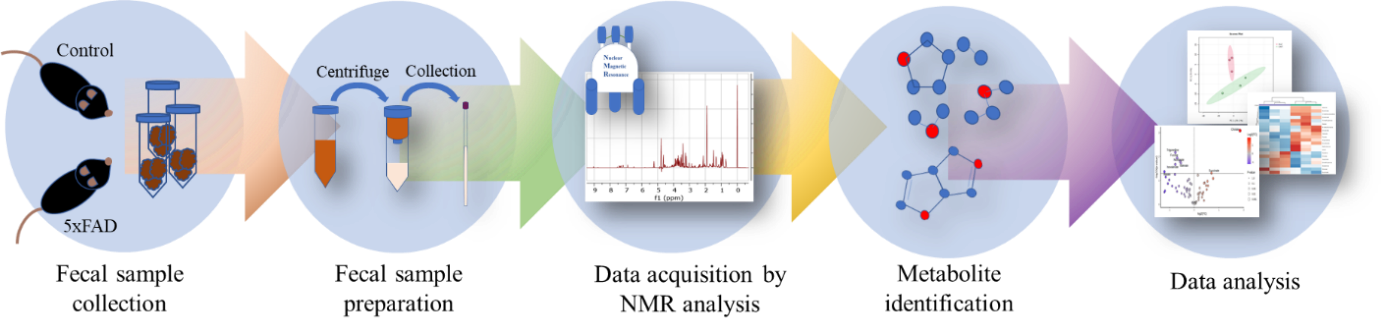
**

Supplement: Supplementary file 2 — Supplementary Material 2 (DOCX 337 KB) [file 11011_2026_1848_MOESM2_ESM.docx]

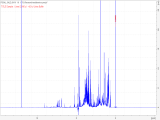

Supplement: Supplementary file 7 — Supplementary Material 7 (DOCX 21.2 MB) [file 11011_2026_1848_MOESM7_ESM.zip › NMR-based_fecal_metabolomics_data_ATB/FECAL_3ALZ_0HYV/10/pdata/1/thumb.png]

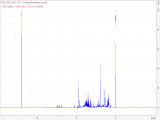

Supplement: Supplementary file 7 — Supplementary Material 7 (DOCX 21.2 MB) [file 11011_2026_1848_MOESM7_ESM.zip › NMR-based_fecal_metabolomics_data_ATB/FECAL_3ALZ_2HYV/10/pdata/1/thumb.png]
