# Supplementary material for: A perspective for alzheimer disease from gut microbiota-associated NMR-based fecal metabolomics: a study with 5XFAD mice
Source: Metab Brain Dis. 2026 Apr 24;41(1):91. doi: 10.1007/s11011-026-01848-2 (PMC13109219; doi:10.1007/s11011-026-01848-2)
Supplement: Supplementary file 6 — Supplementary Material 6 (DOCX 368 KB) [file 11011_2026_1848_MOESM6_ESM.docx]

**Table S1** Identified metabolites in fecal samples of WT and 5XFAD mice and their biological location ( Biological locations of metabolites were supplied from human metabolome database (HMDB, http://www.hmdb.ca/))

| **Metabolite** | **Biological location** |
| --- | --- |
| 1,3-Dimethylurate | BL, FE, U |
| 3-Hydroxyisobutyrate | BL, CSF, FE, S, U |
| 3-Hydroxyisovalerate | BL, CSF, FE, S, U |
| 3-Methylxanthine | BL, FE, U |
| 4-Aminobutyrate | BL, CSF, FE, S, U |
| 4-Pyridoxate | BL, CSF, FE, S, U |
| Acetate | FE, S, U |
| Acetone | BL, BM, BR, CSF, FE, S, U |
| Alanine | BL, CSF, FE, U |
| Arabinose | BL, CSF, FE, U |
| Asparagine | BL, BM, CSF, FE, S, SW, U |
| Aspartate | BL, BM, CSF, FE, S, SW, U |
| Betaine | BL, BM, FE, S, U |
| Butyrate | BL, BM, BR, CSF, FE, S, U |
| Choline | BL, BM, CSF, FE, S, SW, U |
| Creatine | BL, BM, CSF, FE, S, SW, U |
| Creatinine | AF, BL, BM, CSF, FE, S, SW, U |
| Dimethylamine | BL, CSF, FE, S, SW, U |
| Ethanol | BL, CSF, FE, S, SW, U |
| Formate | BL, BM, CSF, FE, S, SW, U |
| Fucose | BL, BM, FE, S, U |
| Fumarate | BL, BM, CSF, FE, S, SW, U |
| Galactose | FE, SW, |
| Glucose | BL, BM, CSF, FE, S, SW, U |
| Glutamate | BL, CC, CSF, FE, S, SW, U |
| Glutamine | BL, BM, CSF, FE, S, SW, U |
| Glycerol | BL, CSF, FE, S, SW, U |
| Glycine | Bile, BL, CSF, FE, S, SW, U |
| Histidine | BL, BM, CSF, FE, S, SW, U |
| Hydroxyacetone | FE, U |
| Isoleucine | BL, BM, CSF, FE, S, SW, U |
| Isopropanol | BL, BR, CSF, FE, S, U |
| Isovalerate | BL, CSF, FE, S, U |
| Lactate | Bile, BL, BM, CC, CSF, FE, S, SW, U |
| Leucine | BL, BM, CSF, FE, S, SW, U |
| Lysine | BL, BM, CSF, FE, S, SW, U |
| Methanol | BL, BM, CSF, FE, S, U |
| Methionine | BL, CSF, FE, S, SW, U |
| Methylamine | BL, FE, S, SW, U |
| N-Acetyltyrosine | BL, U |
| Nicotinate | BL, CSF, FE, S, U |
| p-Cresol | BL, FE, S, U |
| Phenylacetate | BL, CSF, FE, S, U |
| Phenylalanine | BL, BM, CSF, FE, S, SW, U |
| Proline | BL, BM, CSF, FE, S, SW, U |
| Propionate | BL, CSF, FE, S, U |
| Propylene glycol | BL, BR, CSF, FE, S, SW, U |
| Pyruvate | BL, BM, CC, CSF, FE, S, SW, U |
| Riboflavin | BL, CSF, FE, S, U |
| Serine | BL, CSF, FE, S, SW, U |
| Succinate | BL, BM, CSF, FE, S, SW, U |
| Taurine | Bile, BL, BM, CSF, FE, S, U |
| Threonine | BL, BM, CSF, FE, S, SW, U |
| Trigonelline | BL, FE, U |
| Trimethylamine | BL, CSF, FE, S, U |
| Tryptophan | BL, BM, CSF, FE, S, SW, U |
| Tyrosine | BL, BM, CSF, FE, S, SW, U |
| Uracil | AF, BL, CSF, FE, S, U |
| Urocanate | BL, FE, S, SW, U |
| Valerate | BL, BR, FE, S, U |
| Valine | BL, BM, CSF, FE, S, SW, U |
| Xanthine | BL, CSF, FE, S, SW, U |
| Xanthosine | BL, FE, U |
| Trimethylamine N-oxide | BL, CSF, FE, S, U |
| Adenosine | BL, BM, CC, CSF, FE, S, U |
| Malate | BL, CC, CSF, FE, S, SW, U |
| Cholate | Bile, BL, FE, U |

BL; Blood, FE; fecal, U; urine, S; saliva, CSF; cerebral, SW; sweat, BM; breast milk, CC; Cellular Cytoplasm, AF; Amniotic Fluid, BR; Breath

**Table S2** Overview of metabolic pathways associated with all metabolites detected in fecal samples of mice

| **Metabolic Pathway** | ***p*-value** | **Impact value** |
| --- | --- | --- |
| Alanine, aspartate and glutamate metabolism | 6.7239x10^-08^ | 0.6234 |
| Glyoxylate and dicarboxylate metabolism | 3.3827x10^-06^ | 0.11 |
| Pyruvate metabolism | 5.0148x10^-05^ | 0.29918 |
| Valine, leucine and isoleucine biosynthesis | 5.7204x10^-05^ | 0 |
| Glycine, serine and threonine metabolism | 5.8635x10^-05^ | 0.56068 |
| One carbon pool by folate | 1.0634x10^-04^ | 0.18901 |
| Arginine biosynthesis | 7.101x10^-04^ | 0.12234 |
| Butanoate metabolism | 9.458x10^-04^ | 0.03175 |
| Histidine metabolism | 1.2317x10^-03^ | 0.34426 |
| Citrate cycle (TCA cycle) | 2.9849x10^-03^ | 0.153 |
| Phenylalanine metabolism | 3.0832x10^-03^ | 0.35714 |
| Arginine and proline metabolism | 4.6959x10^-03^ | 0.08953 |
| Phenylalanine, tyrosine and tryptophan biosynthesis | 5.6854x10^-03^ | 1 |
| Glycolysis or Gluconeogenesis | 8.003x10^-03^ | 0.12616 |
| Nitrogen metabolism | 1.3645x10^-02^ | 0 |
| Pantothenate and CoA biosynthesis | 2.3422x10^-02^ | 0 |
| beta-Alanine metabolism | 2.6725x10^-02^ | 0 |
| Valine, leucine and isoleucine degradation | 3.5322x10^-02^ | 0.02711 |
| Galactose metabolism | 5.1471x10^-02^ | 0.39113 |
| Neomycin, kanamycin and gentamicin biosynthesis | 6.2484x10^-02^ | 0 |
| Nicotinate and nicotinamide metabolism | 7.9659x10^-02^ | 0 |
| Cysteine and methionine metabolism | 8.4167x10^-02^ | 0.1263 |
| Riboflavin metabolism | 1.2114x10^-01^ | 0.5 |
| Tyrosine metabolism | 1.4567x10^-01^ | 0.16435 |
| Propanoate metabolism | 1.5258x10^-01^ | 0 |
| Primary bile acid biosynthesis | 1.7675x10^-01^ | 0.04478 |
| Purine metabolism | 1.8499x10^-01^ | 0.03018 |
| Lipoic acid metabolism | 2.2215x10^-01^ | 0.0017 |
| Glutathione metabolism | 2.2215x10^-01^ | 0.10839 |
| Taurine and hypotaurine metabolism | 2.2787x10^-01^ | 0.42857 |
| Vitamin B6 metabolism | 2.525x10^-01^ | 0 |
| Porphyrin metabolism | 2.5796x10^-01^ | 0 |
| Biotin metabolism | 2.7636x10^-01^ | 0 |
| Pyrimidine metabolism | 3.5327x10^-01^ | 0.05261 |
| D-Amino acid metabolism | 3.8492x10^-01^ | 0 |
| Starch and sucrose metabolism | 3.8492x10^-01^ | 0.32523 |
| Glycerolipid metabolism | 4.0463x10^-01^ | 0.23676 |
| Fructose and mannose metabolism | 4.4221x10^-01^ | 0 |
| Pentose and glucuronate interconversions | 4.6012x10^-01^ | 0 |
| Ubiquinone and other terpenoid-quinone biosynthesis | 4.6012x10^-01^ | 0 |
| Selenocompound metabolism | 4.7747x10^-01^ | 0 |
| Lysine degradation | 6.2351x10^-01^ | 0 |
| Sphingolipid metabolism | 6.4749x10^-01^ | 0 |
| Glycerophospholipid metabolism | 6.9106x10^-01^ | 0.02569 |
| Tryptophan metabolism | 7.3815x10^-01^ | 0.14305 |
| Amino sugar and nucleotide sugar metabolism | 7.4669x10^-01^ | 0 |


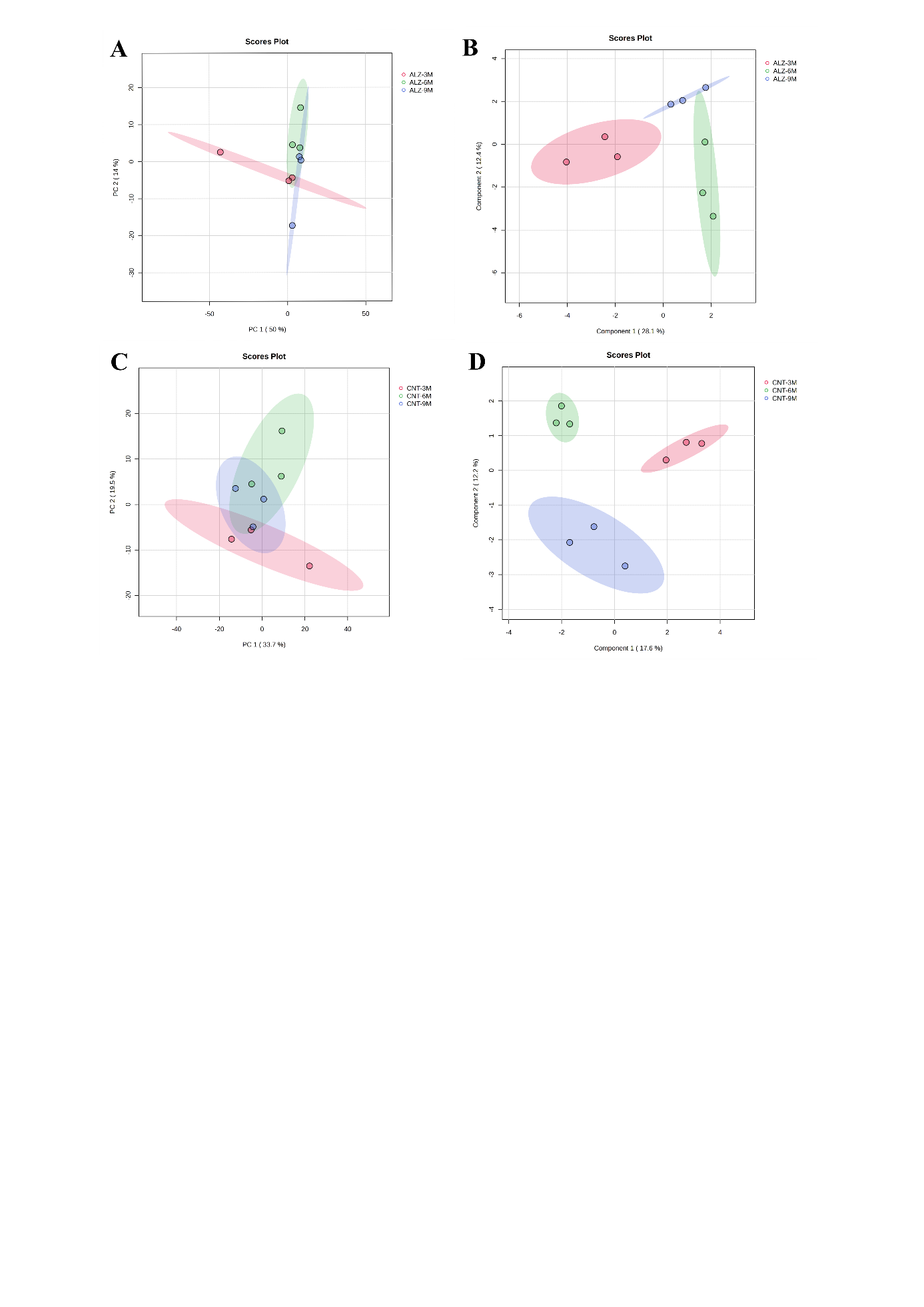


**Fig. S1** The longitudinal comparison of metabolites in three different-aged 5XFAD and healthy mice using PCA score plot (A-C) and sPLS-DA score plot (B-D) according to fecal metabolite profiles. ALZ; 5XFAD mice, CNT; WT mice, 3M; 3-month-old mice and pink in PCA and sPLS-DA, 6M; 6-month-old mice and green in PCA and sPLS-DA, 9M; 9-month-old mice and blue in PCA and sPLS-DA
